# Exploring “phasic” vs. “tonic” accounts of the effect of switch probability on the auditory attention switch cost

**DOI:** 10.1177/17470218241256361

**Published:** 2024-06-10

**Authors:** Amy Strivens, Iring Koch, Aureliu Lavric

**Affiliations:** 1 Institute for Psychology, RWTH Aachen University, Aachen, Germany; 2 Department of Psychology, University of Exeter, Exeter, UK

**Keywords:** Switch cost, switch probability, proportion switches, task-set reconfiguration, attention “cocktail party”

## Abstract

Task-switching experiments have shown that the “switch cost” (poorer performance for task switches than for repetitions) is smaller when the probability of a switch is high (e.g., 0.75) than when it is low (e.g., 0.25). Some theoretical accounts explain this effect in terms of top-down control deployed in advance of the task cue (“pre-cue reconfiguration”). We tested such accounts by manipulating the time available before the onset of the cue (the response-cue interval, RCI), reasoning that top-down pre-cue reconfiguration requires time and therefore its effect should increase with RCI. Participants heard a man and a woman simultaneously speaking number words and categorised the number (< 5 vs. > 5) spoken by the voice specified by a pictorial gender-related cue presented at an RCI of 100 ms or 2,200 ms. The target voice switched with a probability of 0.25 or 0.75 (in separate sessions). In Experiment 1, RTs revealed a large effect of switch probability on the switch cost in the short RCI, which did not increase in the long RCI. Errors hinted at such an increase, but it did not receive clear statistical support and was disconfirmed by a direct and better powered replication in Experiment 2, which fully confirmed the RT pattern from Experiment 1. Thus, the effect of switch probability on the switch cost required little/no time following the response to emerge—it was already at full magnitude at a short RCI—challenging accounts that assume “phasic” deployment of top-down task-set control in advance of the cue.

Performance of a task requires a coherent organisation of cognitive processes—commonly referred to as *task set* (Rogers & Monsell, 1995). The goal-related (intentional, “top-down”) control of task set has been the subject of intense scrutiny, and often such studies have employed the *task-switching paradigm* (for reviews, see Koch et al., 2018; Koch & Kiesel, 2022; Monsell, 2003, 2015). In its most widely used variant, *task cueing* (e.g., Meiran, 1996), participants perform two or more simple tasks in an unpredictable sequence, and the relevant task is specified on each trial by a task *cue* presented simultaneously with, or at some cue-stimulus interval (CSI) before, the imperative stimulus. Such studies almost universally reveal slower responses and higher error rates for task-switches than task-repetitions (see Kiesel et al., 2010; Koch et al., 2018, for reviews). This performance *switch cost* (Rogers & Monsell, 1995) has been found to be strongly modulated by several factors, reflecting separable sources of the switch cost.

In particular, increasing the CSI tends to substantially reduce (though not eliminate) the switch cost (e.g., Meiran, 1996; Monsell & Mizon, 2006; Van’t Wout et al., 2015). This reduction in switch costs with preparation has been widely viewed as the clearest evidence of top-down *task-set reconfiguration* (e.g., Monsell, 2003, 2017; Rogers & Monsell, 1995) that can be performed in advance of the stimulus onset if there is opportunity to do so. Conversely, increasing the interval between the previous response and the current stimulus while keeping the CSI (i.e., the preparation time) constant by extending the response-cue interval (RCI) also tends to reduce the switch cost (e.g., Koch, 2001; Meiran et al., 2000). This effect of RCI on switch costs has been commonly seen as evidence of the dissipation/decay of “passive” task-set persistence, or *task-set inertia* (Allport et al., 1994; see also Koch & Kiesel, 2022; Monsell, 2003).

Converging evidence for advance task-set reconfiguration has been provided by task-switching studies that obtained EEG (e.g., Elchlepp et al., 2012; Karayanidis et al., 2003; Kieffaber & Hetrick, 2005; Lavric et al., 2008) and eye-movement (Longman et al., 2014, 2017, 2021) measures of processing during the CSI. For task-set inertia, there is also evidence based on studies using EEG (Elchlepp et al., 2015; Evans et al., 2015), fMRI (Yeung et al., 2006), and eye-tracking (Longman et al., 2014, 2016, 2017), which documented correlates of brain-activity and eye-movements associated with the no longer relevant task-set.

The present study is concerned with a further factor that has a strong influence on the switch cost—the probability of a switch (also referred to as “proportion of switches” or “list-wide proportion of switches,” e.g., Siqi-Liu & Egner, 2020). A substantial (and growing) body of studies (Dreisbach & Haider, 2006; Geddert & Egner, 2022; Kikumoto et al., 2016; Mayr et al., 2013; Monsell & Mizon, 2006; Siqi-Liu & Egner, 2020; Siqi-Liu et al., 2022) has shown that when a switch has a lower probability than a repetition (e.g., 0.25 vs. 0.75), the switch cost is larger than when switches and repetitions are equiprobable (0.5). Conversely, increasing switch probability to 0.75 results in a substantially smaller switch cost. This effect of switch probability on the switch cost strongly interacts with the preparation interval, such that it is larger, or only present, on short CSI trials (e.g., Mayr et al., 2013; Monsell & Mizon, 2006; Siqi-Liu & Egner, 2020). Effects of switch probability have been documented not only in performance measures (RT and accuracy) but also in eye-movement behaviour—preferential fixating on the target attribute over the non-target attribute is manifested on repeat trials earlier than on switch trials—but this difference is reduced as switch probability is increased and eliminated when it is as high as 0.75 (Mayr et al., 2013; see also Kikumoto et al., 2016).

Recently, we examined the effect of switch probability on the cost of switching auditory attention in a “cocktail party” setting (Strivens et al., 2024). In that study, we employed an auditory attention switching paradigm introduced by Koch et al. (2011), in which participants were cued to switch attention between two simultaneous voices each saying a number. An important feature of the design is that the target voice was the only task-set parameter that switched, whereas all other parameters—in particular, the categorisation of the number spoken by the target voice as smaller vs. larger than 5 and the category-response mapping—remained constant. At first glance, this paradigm may appear quite different from “conventional” task-switching (task-set control) paradigms—where a task switch tends to involve a change in the required categorisation (S-R rules). However, from its earliest conceptualisations, the “task-set” has been proposed to include both a perceptual (attentional) component and a response (S-R) component (e.g., Meiran & Marciano, 2002; Rogers & Monsell, 1995; Rushworth et al., 2002). The first computational model of task switching (Meiran, 2000) distinguished between the “S-Set” (stimulus set), which comprises parameters governing the selection of the relevant perceptual attribute/dimension, and the “R-Set” (response set), which encompasses parameters that implement S-R rules (a subsequent development of this modelling framework, CARIS, Meiran et al., 2008, maintained this distinction). Furthermore, a nontrivial number of studies have kept the response set component of task-set constant to isolate the effects of switching the stimulus-set component in the visual domain (e.g., Geddert & Egner, 2022; Hsieh & Wu, 2010, 2011; Kieffaber et al., 2013; Meiran & Marciano, 2002; Rushworth et al., 2002, 2005), and in the auditory domain of selective attention either to non-speech sounds (e.g., Nolden & Koch, 2017, 2023) or to speech streams (voices) in the “cocktail party” setting (e.g., Koch et al., 2011; Koch & Lawo, 2014; Monsell et al., 2019).

Our recent study mentioned above (Strivens et al., 2024) is part of this strand of studies which have adapted designs and performance measures from task switching to examine the control of perceptual (attentional) parameters of task-set. We were particularly interested in investigating the effect of the probability of a switch in the target voice on the performance switch cost (and its reduction with preparation)—something that had not been previously documented in the “cocktail party setting.” We found this auditory attention switch cost to be substantially larger when switch probability was 0.25 than when it was 0.75 with a short CSI (50 ms or 400 ms), but not with a longer CSI (900 ms or 1,400 ms). Consequently, there was a significant reduction in switch cost with CSI when switch probability was 0.25 and no such reduction when it was 0.75. A similar pattern of interactions between switch probability, switch cost and CSI has been previously documented in “conventional” task-switching experiments where switches involved changes in S-R rules (e.g., Mayr et al., 2013; Monsell & Mizon, 2006). Recently, Geddert and Egner (2022) have confirmed the smaller switch cost for a high (0.75) switch probability than for a low (0.25) switch probability in an experiment that required participants to shift attention between “local” and “global” visual features in Navon stimuli, while keeping the response set constant (the cue that specified the required level attentional focus appeared simultaneously with the stimulus: CSI = 0 ms).^1^ Thus, the robust effects of switch probability on the switch cost extend to perceptual/attentional selection parameters of task-sets and to paradigms where only those parameters switch and are observed mainly for conditions with short CSIs.

Several theoretical accounts of the effect of switch probability on the switch cost have been proposed (e.g., Dreisbach & Fröber, 2019; Dreisbach & Haider, 2006; Kikumoto et al., 2016; Monsell & Mizon, 2006; Musslick & Cohen, 2021; Siqi-Liu et al., 2022). One important distinction (cf., Siqi-Liu et al., 2022) is between *phasic* accounts—which explain the effects of switch probability in terms of relatively brief task-set control processes at the time scale of individual trials (where a “trial” is a single cue-stimulus-response sequence)—and *tonic* accounts—which propose sustained biasing of task-set control, at a time scale of tens or hundreds of trials.^2^

According to the earliest phasic account proposed by Monsell and Mizon (2006), when the probability of a switch is low, task-set reconfiguration is initiated only after the onset of the task cue. Conversely, when the likelihood of a switch is high, task-set reconfiguration may not wait for the task cue—it may be initiated (in anticipation of a likely switch) before the cue on all/most trials, including trials that would eventually turn out to be task repetitions. Monsell and Mizon envisaged that either or both of two forms of such “pre-cue reconfiguration” may occur: (1) preparing for another task and (2) disengaging from the current task, achieving a “neutral” control state. Both forms of pre-cue reconfiguration would improve performance on the following trial if (as anticipated) it turns out to be switch and impair performance if it turns out to be a task repetition, resulting in a smaller switch cost when switch probability is high. An account related (though not equivalent) to the notion of active disengagement was put forward by Kikumoto et al. (2016), who proposed that when switches are relatively rare/unlikely, participants may endogenously maintain the most recent task-set configuration in a state of high activation until the onset of the next task cue, but there is no such “active” task-set maintenance from one trial to another when switch probability is high—resulting for the latter condition in poorer task repetition performance and, possibly, improved task switch performance, hence a smaller switch cost. The above accounts all have in common the notion that some phasic top-down task-set control process occurring before the onset of the task cue is deployed differentially in conditions of low vs. high switch probability. In the two accounts put forward by Monsell and Mizon (2006), this process (preparation or disengagement) is akin to task-set reconfiguration, whereas in the third (Kikumoto et al., 2016), this is a process of “active” maintenance of the task-set configuration from the preceding trial; hence, we will refer to them collectively as “pre-cue (re)configuration” accounts.

In contrast, tonic accounts are framed in terms of sustained (continuous) control biases that are sensitive to switch probability and also to other factors such as rewards associated with task-sets. These accounts have been grounded in the notion that task-set control must achieve an optimal balance (given one’s goal and the environment) between two contradictory (reciprocal) processing metacontrol “modes”—stability vs. flexibility (e.g., Dreisbach & Fröber, 2019; Dreisbach & Haider, 2006; Goschke, 2000; see also Geddert & Egner, 2022). Dreisbach and Fröber (2019) proposed that a low switch probability context encourages stability, whereas a high switch probability context encourages rebalancing towards flexibility (see also Musslick & Cohen, 2021). What processes might achieve such rebalancing? Dreisbach and Fröber suggested two kinds of mechanism for achieving flexibility: lowering the Working Memory (WM) updating threshold (cf., Goschke, 2013), and keeping multiple task-sets active in WM. A further mechanism proposed by Musslick and colleagues (Musslick & Cohen, 2021; Musslick et al., 2018) that may achieve this is the adjustment of the *gain* parameter of the task activation function—a computational implementation of the “commitment” to currently cued task—a lower gain makes it easier to switch to another task, but it also reduces the benefit of repeating the current task, resulting in a smaller switch cost. We return to the discussion of these mechanisms in the General Discussion.

The present study employs the auditory attention (voice) switching paradigm described above, to test the phasic pre-cue (re)configuration accounts of switch probability in an attempt to tip the balance of evidence either in their favour or in favour of alternative accounts of switch probability—such as the tonic accounts above, or other kinds of phasic accounts where the effects of switch probability are not due to effortful top-down control (Siqi-Liu et al., 2022, see General Discussion). What re(configuration) process(es) may the current voice switching paradigm involve? We propose that attentional selection of one of two simultaneous voices requires top-down activation of the auditory attentional/perceptual template for the relevant voice. Such a template likely comprises a representation of the fundamental frequency and/or other characteristics of the target voice, such as the vocal tract length. The substantial performance cost caused by a switch of the target voice (e.g., Koch et al., 2011) and the observation in our previous studies (Monsell et al., 2019; Strivens et al., 2024) that this switch cost can be reduced if the cue is presented sufficiently in advance (at a longer CSI) indicate that switch-related reconfiguration of the attentional template for the target voice is effortful and time-consuming, but may be done in advance of hearing the voices, provided adequate opportunity for preparation. Pre-cue (re)configuration accounts may explain the effect of switch probability on the cost of switching the target voice in our recent study (Strivens et al., 2024) by either: (1) the activation of the attentional template for the previously irrelevant voice (or the deactivation of the template for the previously relevant voice) as soon as a response is made—and before cue onset—in anticipation of a likely switch when the probability of a switch is high, or (2) by active maintenance of the attentional template for the previously-relevant voice only when the probability of a switch is low (see above the discussion of Monsell & Mizon, 2006, and Kikumoto et al., 2016, regarding the first and second possibility, respectively). Tonic accounts can also be articulated in terms of attentional templates. For example, if one subscribes to the notion that a higher switch probability may result in a lower WM updating threshold, or multiple task-sets being held in WM, this can be extended to perceptual components of a task-set, such as the attentional template for a voice—a higher switch probability may result in comparable activation of multiple attentional templates, whereas a lower probability may promote holding a single template in WM.

Previous empirical studies have raised some issues regarding pre-cue (re)configuration accounts. For example, the finding that switch probability influences switch costs not only when participants switch between two tasks but also when they switch among three tasks (e.g., Kikumoto et al., 2016; Mayr et al., 2013; Siqi-Liu & Egner, 2020) is somewhat problematic for the proposal that preparation may start before the task cue when switch probability is high (Monsell & Mizon, 2006), because one would not know for which of two (remaining) tasks to prepare. However, this is not an insurmountable issue—the extra assumption that one could randomly prepare for one of the two alternative tasks would still result in a reduced benefit of a task repetition and an improved performance on half of the switch trials. Furthermore, the number of tasks in play would not affect the other two potential mechanisms of pre-cue (re)configuration: disengagement from (or inhibition of) the most recent task-set in conditions of high switch probability (Monsell & Mizon, 2006) and active task-set maintenance in conditions of low switch probability (Kikumoto et al., 2016).

The current investigation examines a fundamental aspect of the pre-cue (re)configuration accounts above, or indeed of any account that relies on phasic application of top-down control before the onset of the task cue. Top-down task-set control is widely assumed to need time (e.g., Mayr et al., 2013; Meiran, 1996; Monsell, 2003). Indeed, the reduction in switch cost resulting from extending the preparation interval (CSI) is thought to reflect the benefit of extra time available for task-set control processes to be effective in achieving the re-organisation (reconfiguration) of the task set required when the task changes (Monsell, 2003, 2017; Rogers & Monsell, 1995); If top-down control is thus applied before the cue, in particular, for activating (or actively maintaining) the attentional template for the expected voice, it needs sufficient time between the response and the cue to influence the switch cost. Here, we manipulate the time interval available before the cue (the response-cue interval, RCI) and contrast the effect of switch probability on the switch cost in two conditions—one condition where the RCI provides ample opportunity for pre-cue control of auditory attention (i.e., long RCI) vs. another condition where the RCI is too short for effective top-down attentional control (short RCI). To the best of our knowledge, there has not been such a manipulation in the context of the interaction of switch probability and switch costs in the voice-switching literature, or indeed in the wider task-switching literature.

If, as posited by pre-cue (re)configuration accounts, the effect of switch probability on the switch cost (in task switching or auditory attention switching—see our recent study above) is caused by differential deployment of top-down control before cue onset in high vs. low switch probability conditions, then the effect of switch probability on the switch cost should only be present when there is sufficient time available before the cue—in the long RCI condition. Conversely, there should not be an effect of switch probability on the switch cost when the RCI is too short to permit effective deployment of task-set control. Task-switching research (e.g., Koch, 2001; Meiran et al., 2000) and our own auditory attention switching studies (Eben et al., 2020) have shown that longer RCIs tend to reduce the switch cost, possibly by allowing for some “passive” dissipation of the previously selected attentional template (a form of task-set inertia). Since there is no reason for such passive dissipation to be different for our switch probability conditions, we focus here on the effect of RCI on the hypothesised “active” (top-down) pre-cue (re)configuration. As already stated, a short RCI should be insufficient for “active” (re)configuration, hence the difference in switch costs between the high vs. low switch probability conditions should be larger (indeed only present) on long RCI trials.

## Experiment 1

We used the voice switching paradigm which in our recent study (Strivens et al., 2024) yielded robust effects of switch probability on the cost of switching the target voice (see Introduction). As in that study, we manipulated switch probability (0.25 vs. 0.75) within participants, with a separate testing session for each probability to minimise carry-over between the probability conditions. To examine the influence of the time available before the voice cue on the interaction between switch probability and the switch cost, we manipulated the RCI: 100 ms vs. 2200 ms. We kept the preparation interval (CSI) constant and short (50 ms), because the effect of switch probability on the switch cost in previous studies, including ours (Strivens et al., 2024), has been larger (or indeed only present) in conditions with short CSIs.

### Method

#### Participants

The RWTH Aachen University, Faculty 7 (Arts & Humanities) ethics committee approved the procedure for the experiment (approval number: 2020_005_FB7_RWTH AACHEN). We used Prolific (www.prolific.com) to recruit 53 participants, who provided informed written consent to take part in both sessions of the experiment. The requirements for participation were that participants were native English speakers, resided in a predominantly English-speaking country (UK, USA, Canada or Australia) and did not have any uncorrected hearing loss or hearing difficulties. The experiment was made available to users who had reported that they met these criteria in Prolific. These criteria were chosen because the stimuli were English words presented under conditions of high perceptual (energetic) masking from the other speech stream, so unimpaired hearing and highly competent (native-like) English comprehension proficiency were necessary.

The data from three participants who made more than 20% errors were removed because this suggested poor understanding of, or engagement with, the task. Two further participants’ data-sets were removed because their error rate exceeded 3 SDs of the entire sample (which gave a cut-off rate of 9% errors). Of the 48 participants whose data were included in the analysis (24 males, 24 females), 46 had ages between 20 and 70 (*M* = 38.1; *SD* = 13), and two participants did not disclose their age.

#### Statistical power considerations

We are not aware of studies that have tested the interaction between RCI, switch/repeat and switch probability.^3^ Hence, we have followed the recommendations of Brysbaert and Stevens (2018) for power in a repeated measures design. Based on sampling simulations using data from two mega-studies, they concluded that 1,600 observations (in total, over participants and trials) in the smallest analysis cell enable the detection of smaller-than-medium repeated measures effects (0.3–0.4) and a power level of 0.8. The smallest cell of our analysis has 32 observations for each of 48 participants, resulting in 1,536 observations in total. This number of observations is very close to the recommended 1,600, while ensuring the necessary balancing of trials and conditions. In addition, we were also informed by our recent study (Strivens et al., 2024), where the first experiment used the same voice cueing paradigm and the same within-participants manipulations of target voice switch/repetition and switch probability, in conjunction with a temporal manipulation—of the cue-stimulus interval (CSI). That experiment detected both the effect of the probability on the switch cost (two-way interaction) and the modulation of these factors by CSI (the three-way interaction). In the current Experiment 1, there are 50% more (67% more in Experiment 2) observations overall (participants x trials) in the smallest cell of the three-way interaction; hence, the present experiments are considerably better powered for detecting an interaction of this order involving two factors that are identical to those in our previous study (voice switch/repetition and switch probability).

#### Task and materials

The online provider Gorilla Experiment Builder (www.gorilla.sc) was used to host and conduct this experiment. Participants were asked to wear headphones, over which two voices were presented simultaneously (one male and one female). Each voice spoke one of eight possible number words (referring to numbers 1–9, excluding 5) and the participant had to categorise the number word spoken by the target voice specified by a visual cue (see below) as < 5 or > 5. They then responded by pressing the “s” key on the computer keyboard for < 5 and the “k” key for > 5. The voice stimuli were recordings of four speakers: two males and two females. The RWTH Aachen Institute of Technical Acoustics recorded one of the female voices in an anechoic chamber (Loh & Fels, 2020). The other voices were recorded in non-specialist conditions while ensuring that no background noise or echo affected the recordings. For all four speakers, the best utterance of each digit was chosen from three or four instances.

All four possible pairs of voices were used. For each pair, all digit combinations were used to create separate two-talker compounds (except where the two talkers said the same number word). Each of these compounds had a duration of 600 ms, with the first vowel of each number starting at approximately the same point within the recording. The fundamental frequencies and sound intensities were edited so that the frequencies were similar within genders and had a similar variance between genders, while the intensities were similar across all speakers and digits. The four voice pairs were counterbalanced across participants so that participants only experienced one voice pair in both sessions (one session per switch probability condition).

**Figure 1.**
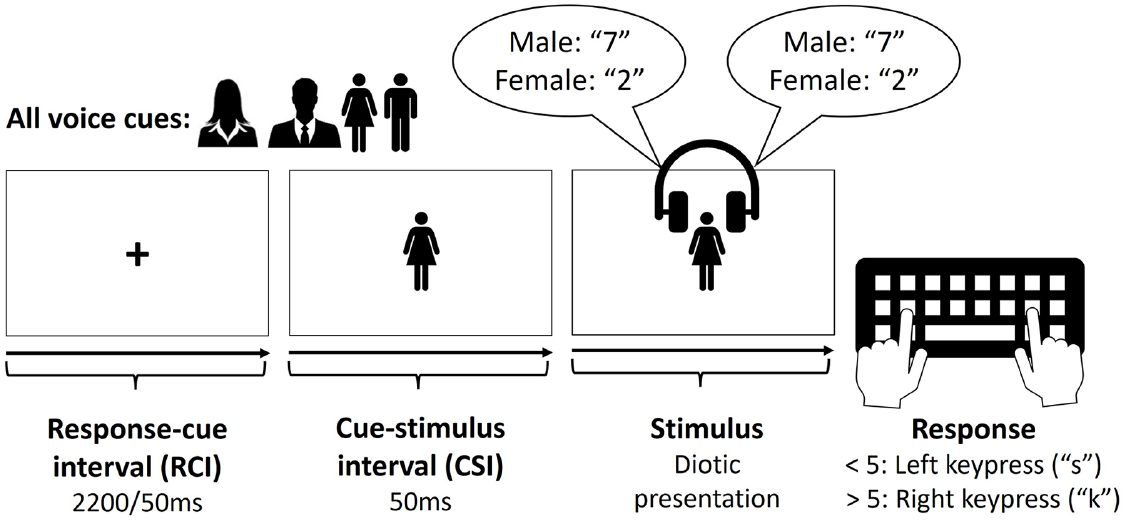
Voice cues and the time-course of one trial.

The cues were semantically transparent pictures, with two cues used for each target gender (one silhouette and one full-body icon, see Figure 1). The type of cue (silhouette or body icon) switched on each trial, irrespective of whether the target voice switched or repeated. This avoided immediate cue repetition and unconfounded switches of target voice from cue switches (cf., Monsell et al., 2019; Monsell & Mizon, 2006; Strivens et al., 2024). The silhouettes and icons had the following pixel dimensions (in parentheses—in mm on a laptop with a 14.2 inch screen): male silhouette 125 x 115 (29 x 27); female silhouette 105 x 115 (25 x 27); male icon 70 x 155 (15 x 36); female icon 82 x 154 (19 x 36). The cue-stimulus interval (CSI) was fixed at 50 ms, with the cue remaining on screen during the CSI and post-stimulus onset, until the participant gave a response. This was entered into Gorilla as a value 50 ms less than the intended CSI (0 ms), because our pilot testing in Gorilla found an average delay of 50 ms when presenting sound files. The response-cue interval (RCI) had a duration of either 100 ms or 2,200 ms, which varied between (but was constant within) blocks (see below). During the RCI, a fixation cross was presented centrally. Participants had 3,000 ms to respond following the stimulus onset; failure to respond within this interval led to “No response” being displayed centrally for 3,000 ms, whereas an incorrect response led to “Error” being displayed centrally for 3,000 ms.

One sequence of trials was created for each switch probability condition in each participant (a total of two sequences per participant) using visual basic scripts. The sequences were subject to a number of constraints, which we outline in what follows (based on the 0.25 switches probability condition; for the 0.75 switch probability condition, the sequence criteria were the same but switches and repetitions were swapped). The trial sequence for each switch probability condition consisted of two sub-sequences (one for each RCI). Each sub-sequence contained 160 trials, of which 25% were switch trials (40 trials) and 75% were repeat trials (120 trials). Of these, 20% were response-congruent (8 switch and 24 repeat) and 80% were response-incongruent (32 switch and 96 repeat). Response (“s” or “k”) and target voice (male or female) were jointly balanced across response congruence and switch/repeat trials, with each congruence and switch/repeat combination having 50/50 female/male voices and each voice 50/50 left-hand/right-hand responses. Using these constraints, it was ensured that the RCI x switch/repeat x response congruence x voice gender x response category combinations were perfectly balanced for each participant. A further important constraint of the trial sequences was that the numbers spoken by the two voices on each trial could never be repeated on the subsequent trial.

On incongruent trials, the eight target numbers were equally likely to be spoken by the male or the female voice for all combinations of RCI x switch/repeat x voice gender. This meant that each number occurred twice on switch trials and six times on repeat trials. On congruent trials, this was the case for 2/3 of the repeat trials. For the remaining 1/3 repeat trials and (separately) for the switch trials, this balancing was achieved across the two RCIs—in one RCI, the male voice (as target voice) spoke numbers 1–4 and the female voice (as target voice) spoke numbers 6–9, and this was reversed in the other RCI. The non-target numbers (spoken by the non-target voice) were balanced as follows. For 2/3 of the incongruent repeat trials, the four possible incongruent numbers occurred equally in each RCI x voice gender combination. For the other 1/3 and (separately) for the incongruent switch trials, the four possible numbers were split randomly so that two were said by the male voice and two by the female voice. The allocation of these numbers to voices in one RCI was then reversed in the other RCI, meaning that on incongruent trials (across RCIs), each target number co-occurred equally often with all possible non-target numbers. The congruent trials were not included in the analysis (see below); hence on these trials, the number spoken by the non-target voice was randomly chosen from the three possible congruent digits.

The two sub-sequences described above (one per RCI) were split into four blocks of 40 trials, two start-up (filler) trials were inserted at the beginning of each of block (see below), and the blocks from the two sub-sequences (RCIs) were alternated, e.g., RCI = 100-Block1, RCI = 2200-Block1, RCI = 100-Block2, RCI = 2200-Block2, RCI = 100-Block3, RCI = 2200-Block3, RCI = 100-Block4, RCI = 2200-Block4. The order of RCIs, which for a given participant was the same in the two switch probability conditions (testing sessions), was counterbalanced over participants. At least one start-up (filler) trial that cannot be included in the analysis was required at the beginning of each block, because the first trial of a block is unclassifiable as switch vs. repetition. We inserted a second start-up filler trial because in previous auditory online experiments using Gorilla participants sometimes reported not hearing well the auditory stimulus on the first trial of a block. The voice on the second start-up trial (preceding the first to-be-analysed trial) was selected depending on the voice and switch/repeat condition on the subsequent trial, whereas the numbers spoken by the target and non-target voices (and the corresponding response categories) were selected randomly, subject to the above-mentioned constraint that the numbers could not be repeated from one trial to the next. On the first start-up trial, the voices and numbers were all selected randomly, subject to the same constraint.

#### Procedure

The experiment was run across two sessions (one per switch probability condition), each lasting ~35 min and separated by a minimum of 24 h. A headphone check by Milne et al. (2021), available as an open-access material on Gorilla.sc, was completed by participants before each session to ensure that participants were using headphones with an adequate sound quality for the experiment. This was followed by two practice phases. The “single-voice phase” familiarised participants with the voices they would hear during the experiment, the cues they would see, and the categorisation task they would have to perform. During the single-voice phase, only one voice was presented on each trial across three 16-trial blocks. The first block presented the male voice in isolation, the second presented the female voice in isolation, and the third presented the two voices in random order. During the single-voice practice phase, the RCI was 2,200 ms and CSI was 50 ms. This was followed by the second practice phase, which included four practice blocks of 25 trials (two for each RCI) where the voices were presented simultaneously (as in the main blocks). The probability of a voice switch during the second practice phase was the same as in the main part of the session.

The instructions for the main part of the experiment were subsequently presented and informed participants that the task would be the same as in the second practice phase (in longer blocks). Participants were informed of the switch probability in the current session, e.g., except from instructions from the 0.25 switch probability session: “The current session has 25% switch trials. Therefore, in the following practice blocks and main blocks, you will notice that the voice to attend to will remain the same more often than it changes.” Participants were also told about the performance-related monetary bonus (see below). The main phase consisted of eight blocks of 42 trials (a total of 336 trials), each block containing quarter of a sequence of trials for one RCI (40 trials), as explained above, plus two response-incongruent start-up trials subsequently excluded from the analysis.

#### Performance-related monetary bonus

To maximise participant engagement with the task, a performance score was calculated starting from the second practice phase for each block using the formula: mean RT/10 + number of errors x 5. After each block, a target score was calculated by averaging the scores for the previous blocks of that RCI (including the relevant blocks from the second practice phase). Upon completion of the second practice phase, participants were informed that, from then on, they would receive a bonus of 30 pence (GPB 0.3) each time they improved their target score. Participants were informed of their score for the previous block, whether this beat the target score (awarding 30 pence) and their new target score to beat after each of the main blocks. At the end of each session, the total number of bonus points and their translation into monetary value was presented to the participants.

#### Data processing

We excluded trials where Gorilla recorded loading delays > 10 s between the response and the fixation cross of the following trial, and where the CSI was more than 70 ms longer than planned, or the RCI was more than 50 ms longer than planned—such problems were likely due to fluctuations of the internet connection. A total of five trials, which would have been otherwise included in the analyses, had to be excluded for these reasons over all 48 participants.

Following the approach of Monsell et al. (2019), the analysis was restricted to response-incongruent trials (80%) from the main part of the experiment (excluding practice), because on congruent trials, participants could potentially respond above chance without selectively attending to the target voice. The two start-up trials from each block were excluded from the RT and error analyses, as were the trials following errors, and trials where RT < 200 ms. In the RT analyses, error trials were also excluded.

#### Design and statistical analysis

The mean RTs and error rates (dependent variables) were submitted to repeated measures ANOVAs with factors (independent variables) SwitchProb (switch probability with 2 levels), Switch/Repeat (2) and RCI (2). Where violations of sphericity occurred, the Huynh-Feldt correction was used (but uncorrected *dfs* are reported). In follow-up ANOVAs, main effects and interactions were reported as statistically significant only if they survived the Bonferroni correction for multiple comparisons (but *p* statistics are reported because these are often ranges rather than precise values, e.g., *p* < .001). Because the critical interaction between SwitchProb, Switch/Repeat and RCI was non-significant for RTs, and given that conventional (frequentist) statistics cannot provide evidence for the null hypothesis, we complimented the ANOVAs with Bayesian analyses of this interaction, as well as of another key interaction—between the SwitchProb and Switch/Repeat at the short RCI—this RCI should be insufficient for time-consuming pre-cue reconfiguration; hence, one would not expect an effect of switch probability on the switch cost for the short RCI based on pre-cue reconfiguration accounts (see Introduction). SPSS v.27.0.1.0 (IBM) was used to conduct the repeated measures ANOVAs and the BayesFactor package in R was used to run the Bayesian analyses.

### Results

#### Reaction times

**Figure 2.**
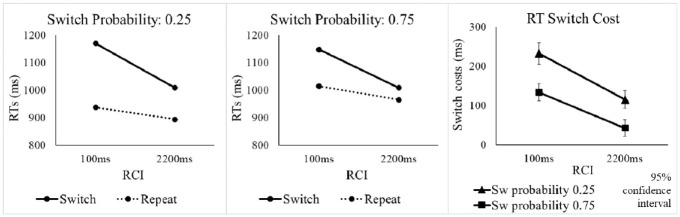
RTs and RT switch costs in Experiment 1 as a function of switch probability, RCI, and switch vs. repetition of the target voice. Here and in all the subsequent figures, error bars show 95% confidence intervals around the mean switch cost.

The omnibus ANOVA of SwitchProb (switch probability), RCI, and Switch/Repeat revealed a significant switch cost, main effect of Switch/Repeat, *F*(1, 47) = 222.58, *p* < .001, η_p_^2^ = .826, and improved overall performance with increasing RCI, main effect of RCI, *F*(1, 47) = 72.12, *p* < .001, η_p_^2^ = .605 (see Table 1 for means). Extending the RCI also led to a reduction in RT switch costs, significant RCI x Switch/Repeat interaction, *F*(1, 47) = 93.17, *p* < .001, η_p_^2^ = .665. The RT switch cost was substantially larger for the 0.25 switch probability condition (see Figure 2), as indicated by the significant SwitchProb x Switch/Repeat interaction, *F*(1, 47) = 100.88, *p* < .001, η_p_^2^ = .682. Follow-up SwitchProb x RCI ANOVAs examining whether this effect of switch probability on the switch cost originated on switch trials or repeat trials (or both) revealed a significant main effect of SwitchProb for repeat trials, *F*(1, 47) = 20.97, *p* < .001, η_p_^2^ = .308, but not for switches (*F* < 1), indicating that the effect of switch probability on the RT switch cost was primarily driven by the lower performance on repeat trials when switch probability was high.

**Table 1.** Mean RT (ms) in Experiment 1 as a function of switch probability, RCI, and switch vs. repetition of the target voice.

		Switch/Repeat			
		Switch		Repeat	
Switch probability	RCI	*M*	*SD*	*M*	*SD*
**0.25**	**100** **ms**	1,171	170	939	146
	**2,200** **ms**	1,010	158	895	126
**0.75**	**100** **ms**	1,149	184	1,015	162
	**2,200** **ms**	1,010	169	966	142

The crucial SwitchProb x Switch/Repeat x RCI interaction in the omnibus ANOVA did not approach significance, *F*(1, 47) = 2.63, *p* = .112, η_p_^2^ = .053, revealing no detectable influence of RCI of the effect of switch probability on the switch cost. Since this non-significant frequentist interaction test cannot provide conclusive evidence for the null and against an increase in the effect of switch probability on the switch cost with a longer RCI, we conducted a Bayesian test of this interaction. As can be seen in Figure 2 (rightmost panel), the difference in switch costs between the two probability conditions did not increase in the longer RCI even numerically. In fact, this effect was non-significantly larger in the short RCI. To ensure that in the Bayesian analysis this numerical difference in the opposite direction to that predicted by pre-cue reconfiguration is not treated as evidence for the effect of RCI predicted by pre-cue reconfiguration, we conducted a one-tailed (one-sided) Bayesian t-test, which assessed the evidence specifically for an *increase* in the difference in switch costs between the two probability conditions in the long RCI (as predicted by pre-cue reconfiguration) against the null.^4^ The Bayesian t-test resulted in a Bayes factor (BF_10_) of 0.064, which, according to the Bayes factor classification of Lee and Wagenmakers (2013), provides strong evidence for the null—against a greater effect of switch probability on the switch cost in long RCI than in the short RCI.

Importantly, a large difference in RT switch costs is already evident at the short RCI (see Figure 2). The ANOVA with factors SwitchProb and Switch/Repeat for the short RCI revealed a significant SwitchProb x Switch/Repeat interaction, *F*(1, 47) = 71.76, *p* *<* .001, η_p_^2^ = .604. This interaction was confirmed by a one-sided Bayesian t-test that tested for a larger switch cost in the 0.25 switch probability condition than in the 0.75 switch probability condition at the short RCI, which revealed a BF_10_ = 3.856 x 10^8^, providing overwhelmingly strong evidence for a larger switch cost in the 0.25 probability condition than in the than in the 0.75 switch probability condition at this RCI.

#### Error rates

**Figure 3.**
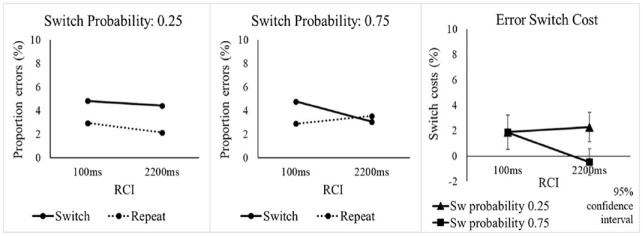
Percentage error rate and error switch cost in Experiment 1 as a function of switch probability, RCI, and switch vs. repetition of the target voice.

The omnibus ANOVA on error rates with the same three factors as for RTs found a significant switch cost, *F*(1, 47) = 19.73, *p* < .001, η_p_^2^ = .296 (see Table 2), as well as a significantly larger switch cost in the low switch probability condition (see Figure 3), as indicated by the SwitchProb x Switch/Repeat interaction, *F*(1, 47) = 5.52, *p* = .023, η_p_^2^ = .105. As for RTs, we conducted follow-up SwitchProb x RCI ANOVAs to investigate whether the effects in the error rates were driven by the switch or repeat trials (or both). For switch trials, there were no significant effects involving SwitchProb, whereas the analysis of repetitions showed an interaction of SwitchProb and RCI which approached significance, *F*(1, 47) = 4.27, *p* = .044 (Bonferroni-corrected *p* = .088), η_p_^2^ = .083. As with RTs, the effects of SwitchProb on error switch costs seemed to be driven mainly by higher error rates on voice repetition trials when the switch probability was higher, especially in the long RCI.

**Table 2.** Error rate (%) in Experiment 1 as a function of switch probability, RCI, and switch vs. repetition of the target voice.

		Switch/ Repeat			
		Switch		Repeat	
Switch probability	RCI	*M*	*SD*	*M*	*SD*
**0.25**	**100** **ms**	4.82	4.84	2.94	2.12
	**2,200** **ms**	4.42	4.16	2.14	1.73
**0.75**	**100** **ms**	4.77	4.12	2.90	3.47
	**2,200** **ms**	3.06	2.39	3.54	3.63

Returning to the omnibus ANOVA, the key interaction of SwitchProb, Switch/Repeat and RCI was significant, *F*(1, 47) = 4.94, *p* = .031, η_p_^2^ = .095, suggesting that the effect of switch probability on the switch cost was influenced by RCI. However, the one-sided Bayesian t-test conducted (as in the RT analysis) for this interaction provided only “anecdotal” evidence (BF_10_ = 2.874) against the null, suggesting caution in interpreting this effect as clear support for pre-cue reconfiguration.^5^

### Discussion

**Figure 4.**
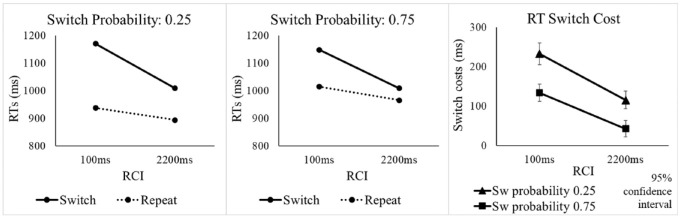
RTs and RT switch costs in Experiment 2 as a function of switch probability, RCI, and switch vs. repetition of the target voice.

As expected based on previous task-switching studies and on our recent investigation of auditory attention switching between simultaneous voices (see Introduction), Experiment 1 revealed substantial effects of switch probability on the switch cost, providing a robust baseline effect, whose modulation by RCI could be examined. We reasoned that if pre-cue (re)configuration is the source of the difference in switch cost between the switch probability conditions, this difference should be present only in the RCI = 2200 ms condition, which provides sufficient time. Although errors indeed seemed to reveal this pattern (*p* = .031 in the crucial three-way ANOVA interaction), albeit only “anecdotally” in the Bayesian test, there was no sign of such a pattern in RTs—where the longer RCI did not result in a larger effect of switch probability on the switch cost, even numerically (see Figure 4) and where the Bayesian test provided strong evidence against a larger difference in switch costs between the switch probability conditions in the long RCI than in the short RCI. Moreover, there was a large and significant effect of switch probability, confirmed by the Bayesian test, on the RT switch cost for the short RCI. At the very least, this indicates that time-consuming pre-cue (re)configuration is not sufficient to explain the effects of switch probability.

The apparent contradiction between the patterns of results in the RTs and errors, along with the uncertainty regarding the diagnostic three-way interaction in the error analysis, in the context of the novel manipulation of RCI, were all strong reasons for conducting a direct replication—which we did in Experiment 2.

## Experiment 2

Experiment 2 was a direct replication of Experiment 1, but with improved statistical power thanks to a 33% increase in the sample size. The aim was to see which pattern of results from Experiment 1 is replicated—the pattern of RTs or the pattern of errors, or both.

### Method

#### Participants

A new sample of 65 participants was recruited via Prolific (using the same inclusion criteria as in Experiment 1, see above). Participants provided informed written consent to participate in the two-session experiment whose procedure was approved by the RWTH Aachen University, Faculty 7 (Arts & Humanities) ethics committee (approval number: 2020_005_FB7_RWTH AACHEN). The data from one participant who made more than 20% errors was removed because this suggested poor understanding of, or engagement with, the task. Of the 64 participants whose data were included in the analysis (33 males, 30 females, 1 other), 60 had ages between 18 and 70 (*M* = 36.2; *SD* = 11.6); four did not disclose their age.

All aspects of the design, materials, task, procedure and data processing were identical to those in Experiment 1.

#### Statistical power considerations

Given the uncertainty regarding the diagnostic interaction between switch probability, switch cost and RCI in the error analyses in Experiment 1 (it was marginally significant in the ANOVA, but only “anectodal” in the Bayesian test), we sought to augment the statistical power by increasing the number of participants. Optimal balancing of design factors over participants (the order of the 0.75 and 0.25 switch probability conditions tested in separate sessions; the order of RCIs over blocks) and stimuli (four pairs of voices) required a number of participants that was a multiple of 16 (two RCI orders x two session orders x four voice pairs). Thus, we increased the target number of participants from 48 (Experiment 1) to 64. This 33% increase in sample size resulted in an increase in the total of observations (participants x trials) from 1,536 to 2,048, which surpasses considerably the 1,600 observations recommended by Brysbaert and Stevens (2018) and provides more than adequate power to detect a smaller-than-medium-sized effect.

### Results

#### Reaction times

The omnibus ANOVA with factors SwitchProb, RCI, and Switch/Repeat revealed a significant switch cost, main effect of Switch/Repeat, *F*(1, 63) = 320.91, *p* < .001, η_p_^2^ = .836, and a significant improvement in overall performance with increasing the RCI, *F*(1, 63) = 38.13, *p* < .001, η_p_^2^ = .377 (see Table 3). As in Experiment 1, there was a significant SwitchProb x Switch/Repeat interaction, *F*(1, 63) = 85.51, *p* < .001, η_p_^2^ = .576, which reflected a substantially larger switch cost for the 0.25 switch probability condition (see Figure 4). When examining the switch and repeat trials in separate SwitchProb x RCI ANOVAs, it was again found (as in Experiment 1) that the effect of switch probability arose primarily from the effect of switch probability on repetition performance (worse performance for the high switch probability), as indicated by the significant main effect of SwitchProb for repetitions, *F*(1, 63) = 25.55, *p* < .001, η_p_^2^ = .289, but not switches (*F* < 2). The RCI x Switch/Repeat interaction was also significant, *F*(1, 63) = 117.34, *p* < .001, η_p_^2^ = .651, reflecting a reduction in switch cost with an increasing RCI.

**Table 3.** Mean RT (ms) in Experiment 2 as a function of switch probability, RCI, and switch vs. repetition of the target voice.

		Switch/Repeat			
		Switch		Repeat	
Switch probability	RCI	*M*	*SD*	*M*	*SD*
**0.25**	**100** **ms**	1,188	163	943	130
	**2,200** **ms**	1,057	176	931	158
**0.75**	**100** **ms**	1,160	160	1,016	149
	**2,200** **ms**	1,038	180	993	169

As in Experiment 1, the critical ANOVA interaction between SwitchProb, RCI, and Switch/Repeat did not approach significance, *F*(1, 63) = 1.56, *p* = .216, η_p_^2^ = .024; the Bayesian t-test provided strong evidence for the null and against a greater effect of probability on the switch cost as RCI increased, BF_10_ = 0.065. The difference in switch cost between the two probability conditions was already large at the short RCI, as indicated by the significant two-way interaction in the SwitchProb x Switch/Repeat ANOVA for this RCI, *F*(1, 63) = 56.12, *p* < .001, η_p_^2^ = .471. This was confirmed by the one-sided Bayesian t-test for this interaction, which provided overwhelmingly strong evidence in support of the presence of a difference in switch costs between the two probability conditions for the short RCI, and against the null, BF_10_ = 7.444 x 10^7^.

**Figure 5.**
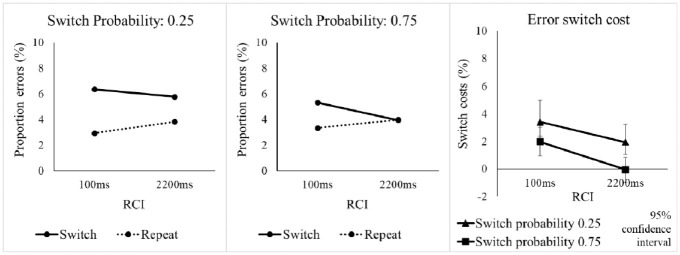
Percentage error rate and error switch costs in Experiment 2 as a function of switch probability, RCI, and switch vs. repetition of the target voice.

The omnibus ANOVA for error rates found a significant switch cost, main effect of Switch/Repeat, *F*(1, 63) = 19.09, *p* < .001, η_p_^2^ = .233 (see Table 4), which interacted significantly with SwitchProb, *F*(1, 63) = 7.06, *p* = .010, η_p_^2^ = .101, with the switch cost being larger in the 0.25 switch probability condition (see Figure 5). Follow-up SwitchProb x RCI ANOVAs for switch and repeat trials separately found a significant main effect of SwitchProb, *F*(1, 63) = 6.97, *p* = .010, η_p_^2^ = .100, for the switches, but not repetitions (*F* < 1).

**Table 4.** Error rate (%) in Experiment 2 as a function of switch probability, RCI, and switch vs. repetition of the target voice.

		Switch/Repeat			
		Switch		Repeat	
Switch probability	RCI	*M*	*SD*	*M*	*SD*
**0.25**	**100** **ms**	6.37	6.82	2.95	2.59
	**2,200** **ms**	5.79	5.23	3.85	3.01
**0.75**	**100** **ms**	5.34	4.71	3.36	3.45
	**2,200** **ms**	3.96	3.68	3.99	3.20

#### Error rates

Crucially, the diagnostic SwitchProb x Switch/Repeat x RCI interaction did not approach significance, *F*(1, 63) = 0.38, *p* = .538, η_p_^2^ = .006. The one-sided Bayesian t-test for this interaction found moderate evidence for the null and against the increase in the effect of switch probability on the switch cost with extending the RCI, BF_10_ = 0.239. To determine whether, as in the RTs, the difference in switch cost between the two probabilities was already present at the short RCI, we tested the SwitchProb x Switch/Repeat interaction for RCI = 100 ms—it approached, but did not reach, significance, *F*(1, 63) = 3.00, *p* = .088, η_p_^2^ = .045, and the Bayesian t-test found weak (anecdotal) evidence for a difference between switch costs between the switch probability conditions for the short RCI, BF_10_ = 1.067.

### Discussion

Experiment 2 confirmed the robust overall effects of switch probability on the RT and error switch costs. With regard to its primary aim of clarifying the diverging patterns of RT and error results in Experiment 1, the pattern of results for RTs remained unchanged—switch costs were again significantly different for the two switch probabilities at RCI = 100 ms and this difference did not (even numerically) increase for RCI = 2,200 ms. Importantly, the lack of an increase in the effect of probability on the switch cost with extending the RCI and the difference in switch cost between switch probabilities of 0.25 and 0.75 at the short RCI both received very strong support from the Bayesian tests. In the errors, the three-way interaction of switch probability, RCI, and switch/repeat no longer approached significance in this better-powered replication, suggesting that the marginal ANOVA interaction in Experiment 1 was likely a false positive; the moderate support for the null in the Bayesian test is consistent with this interpretation. Based on these new findings, it appears that pre-cue (re)configuration is neither sufficient nor necessary to explain the patterns of results in either dependent variable (RTs or errors). We will discuss what this means for the various accounts of the effect of switch probability on the switch cost in General Discussion.

Before turning to other accounts, we ought to consider a less interesting explanation—run length. In cued task switching (e.g., Monsell et al., 2003) and voice-cueing (Monsell et al., 2019), performance is known to improve beyond the 2nd position in the run (first repetition) of a task/target voice. This may explain the larger RT switch cost in the 0.25 switch probability condition, because, by design, this condition contained longer runs of a target voice. Hence, we submitted the RTs from Experiment 2 to an extra analysis limited run positions to 1–2 (the switch and the first repetition). This analysis revealed the same pattern of results as in the main RT analysis—a significant SwitchProb x SwitchRepeat interaction both in the omnibus ANOVA, *F*(1, 63) = 39.48, *p* < .001, ηp^2^ = 0.385, and in the ANOVA restricted to RCI = 100, *F*(1, 63) = 24.29, *p* < .001, ηp^2^ = 0.278, and no significant 3-way interaction in the omnibus ANOVA, *F*(1, 63) = 0.48, *p* = .490, η_p_^2^ = .008. Thus, differences in run length between the switch probability conditions cannot explain out the difference in RT switch cost between these conditions in Experiment 2.

## General discussion

The robust effect of task switch probability on the performance switch cost has been the subject of considerable empirical scrutiny and theorising (see Introduction); recently it has also been documented in a variant of the task-cueing paradigm where the only task attribute that switches is the relevant voice in a multitalker compound (Strivens et al., 2024, see Introduction). Here, we set out to examine three theoretical accounts of the influence of switch probability on the switch cost—all based on a “phasic” control processes that occur early, before the onset of the task cue: preparation of an alternative task-set when the probability of a switch is high (Monsell & Mizon, 2006), disengagement from (inhibition of) the previously-relevant task-set when the probability of a switch is high (Monsell & Mizon, 2006), and active maintenance of the previously-relevant task-set in conditions of low (but not high) switch probability (Kikumoto et al., 2016). The central tenet of all these accounts is that the source of the effect of switch probability on the switch cost is the same kind of phasic, effortful, time-consuming, task-set (re)configuration that occurs after cue onset (during the CSI) in task-cueing experiments (typically leading to a reduction in switch cost with increasing the CSI), but which also occurs before the cue onset depending on switch probability. We tested this core assumption using a novel manipulation in the context of investigating switch probability—the manipulation of the time available before cue onset (RCI). Our reasoning is that (re)configuration can be effective only if there is sufficient time for it before the cue—thus, switch probability should have a greater effect on the switch cost when the RCI is ample than when it is short. Furthermore, if pre-cue (re)configuration is the only (or the primary) source of the effect of switch probability on the switch cost, there should be no difference in switch cost between high vs. low switch probability conditions when the RCI is (too) short.

Contrary to pre-cue reconfiguration, RTs revealed a robust effect of switch probability on the switch cost even for the short RCI and found no increase in this effect when RCI was extended to 2,200 ms (the latter null effect received strong support from the Bayesian tests). The pattern of errors in Experiment 1 appeared consistent with pre-cue (re)configuration—the effect of switch probability on the error switch cost seemed larger (and indeed present only) for the long RCI. However, the uncertainty regarding the crucial ANOVA interaction of switch probability, switch cost, and RCI, for which Bayesian test provided only “anecdotal” evidence, prompted us to conduct a better-powered direct replication in Experiment 2. Experiment 2 confirmed entirely the pattern of RT results from Experiment 1: there was again clear evidence (both from ANOVAs and Bayesian tests) for the presence of a difference in switch cost between the two switch probability conditions when RCI was short, and for the absence of an increase in this difference when RCI was extended. For the errors, the diagnostic three-way ANOVA interaction (see above) no longer approached significance, and the Bayesian test of this interaction provided moderate evidence for the null (no increase in the effect of switch probability on the switch cost as RCI is extended). Thus, the pattern of errors in Experiment 2 was more consistent with the RT results in both experiments than with the error results in Experiment 1. In sum, the results speak against phasic time-consuming attentional control processes, such as the activation (or active maintenance) of the attentional template for the expected target voice, or the de-activation of the attentional template for the voice expected to be no longer relevant, as likely sources of the effect of switch probability on the switch cost.

Our results are consistent with tonic (sustained) control accounts of switch probability. These have tended to be framed in terms of adjustments along the stability-flexibility continuum (Dreisbach & Fröber, 2019; Dreisbach & Haider, 2006; Goschke, 2000). Three variants of such accounts have been outlined in the Introduction—the updating of WM thresholds (e.g., Dreisbach & Fröber, 2019), the adjustment of the gain of the task-set activation function (Musslick & Cohen, 2021), and the concurrent uploading of multiple task-sets into WM (Dreisbach & Fröber, 2019). A further tonic account put forward by Liu and Yeung (2020) based on their analyses of the EEG alpha-band is that low switch probability conditions may encourage the adoption of a sustained “task-set shielding” processing mode. However, tonic control accounts face their challenges. One challenge is that explanations in terms of a global parameter common to all task-sets in play (adjusting the WM threshold or the gain of the task-set activation function) may find it difficult to account for evidence that switch probability seems to have an effect only on the tasks where the probability is manipulated and not on other tasks in play where switches and repetitions are equiprobable (“transfer tasks,” Siqi-Liu & Egner, 2020). Furthermore, most tonic accounts predict that a shift from stability towards flexibility should make the relevant task-set more vulnerable to interference from irrelevant task-sets. Yet, a recent investigation by Geddert and Egner (2022) who used the response congruence effect as a measure of task-set interference found little evidence that a higher switch probability resulted in a larger congruence effect, although it did, as one would expect, result in a smaller switch cost. We note, however, that the congruence effect arises from several sources and does not only reflect task-level interference (Forrest et al., 2014; Steinhauser & Hübner, 2009). Hence, it would be informative to develop measures that isolate task-level interference more specifically.

There is a further account that may be compatible with our results (e.g., Siqi-Liu et al., 2022)—according to which exposure to a high switch probability results in learning-related modulation of the cue encoding, such that the presentation of the cue results in a weaker activation of the task-set if the task-set is the same on the preceding trial (a task repetition)—which also accounts for most of the reported effects of switch probability on the switch cost arising on task repetition trials (the latter was also the case in the present experiments, except for the errors in Experiment 2). Since, according to this account, the “action” takes place following the cue onset, it is compatible with the large effect of switch probability on the RT switch cost in our short RCI condition and with the lack of detectable RCI x switch probability x switch cost interaction in the RTs in both experiments and the errors in Experiment 2.

On a different note, the present results also provide insights on the time course of dissipation of the auditory attentional set—the attentional “template” for the target voice. Koch and Lawo (2014) manipulated the RCI in a voice switching paradigm similar to ours (though without a manipulation of switch probability, which was 0.5 throughout) to examine the influence of RCI on the cost of switching attention between simultaneously presented voices. They found that increasing the RCI up to 1,000 ms did not reduce the switch cost. Yet, when Eben et al. (2020) extended the long RCI to 1,900 ms, they found a reliable reduction in switch cost. In line with this, our long RCI of 2,200 ms clearly reduced the switch cost relative to the RCI of 100 ms (for both probability conditions), which confirms that attentional templates for speech may have greater persistence and require non-trivial time to dissipate, presumably because the processing of speech (and possibly other kinds of auditory information) is serial. In comparison, in visual task-switching experiments (much) smaller increases in RCI (e.g., 132 ms to 532 ms, Meiran et al., 2000; 100 to 1,000 ms, Horoufchin et al., 2011) lead to significant reductions in switch costs.

In conclusion, the present experiments based on a manipulation of the time available before the cue onset provide evidence against existing accounts of the effect of switch probability on the switch cost in terms of a phasic top-down control deployed before the onset of the cue. This evidence can be accounted for in terms of tonic (sustained) adjustments in top-down control, which are not confined to specific intervals of a trial, and in terms of phasic accounts where the locus of the effect of switch probability is after the onset of the cue.

## Notes

1. We revisit this study in General Discussion vis-à-vis its other manipulations and findings.

2. We note that the phasic vs. tonic dichotomy that we used to present theoretical accounts of the effect of switch probability on the task switch cost may seem, at first glance, analogous to the dichotomy between “proactive” vs. “reactive” control (Braver, 2012), which have been used to refer, respectively, to sustained control biases vs. control processes that vary trial to trial. However, “reactive control” has also been used to refer to control driven by exogenous, stimulus-related, variables (e.g., Green, 1998)—which is not consistent with the preparatory pre-stimulus phasic control that we are testing. Hence, the terms “tonic” and “phasic” seem more suitable for presenting the theoretical accounts which we tested and are already used in the literature on the effects of switch probability on the switch cost (e.g., Siqi-Liu et al., 2022).

3. We are also not aware of software applications that allow straightforward estimation of the required sample size (given a specified significance threshold, effect size, and power level) for an interaction between three repeated measures factors. In particular, as far as we are aware, this cannot be done in G*Power (Faul et al., 2007).

4. The fact that each of the three factors in our design had only two levels allowed us to collapse the 3-way interaction into a *t*-test, by computing the switch cost (switch-repeat difference), then the difference in switch costs between the two probability conditions for each RCI—these were submitted to the Bayesian t-test.

5. To anticipate, this interaction was also not replicated for the better powered Experiment 2.

## References

[bibr1-17470218241256361] Allport A. Styles E. Hsieh S. (1994). Shifting intentional set: Exploring the dynamic control of tasks. In Umilta C. Moscovitch M. (Eds.), Attention and performance XV (pp. 421–452). The MIT Press.

[bibr2-17470218241256361] Braver T. S. (2012). The variable nature of cognitive control: A dual mechanisms framework. Trends in Cognitive Sciences, 16(2), 106–113. 10.1016/j.tics.2011.12.010 22245618 PMC3289517

[bibr3-17470218241256361] Brysbaert M. Stevens M. (2018). Power analysis and effect size in mixed effects models: A tutorial. Journal of Cognition, 1(1), 1–20. 10.5334/joc.10 PMC6646942 31517183

[bibr4-17470218241256361] Dreisbach G. Fröber K. (2019). On how to be flexible (or not): Modulation of the stability-flexibility balance. Current Directions in Psychological Science, 28, 3–9. 10.1177/096372141880003

[bibr5-17470218241256361] Dreisbach G. Haider H. (2006). Preparatory adjustment of cognitive control in the task switching paradigm. Psychonomic Bulletin & Review, 13(2), 334–338. 10.3758/BF03193853 16893004

[bibr6-17470218241256361] Eben C. Koch I. Jolicoeur P. Nolden S. (2020). The persisting influence of unattended auditory information: Negative priming in intentional auditory attention switching. Attention, Perception, & Psychophysics, 82, 1835–1846. 10.3758/s13414-019-01909-y 31898070

[bibr7-17470218241256361] Elchlepp H. Lavric A. Mizon G. A. Monsell S. (2012). A brain-potential study of preparation for and execution of a task-switch with stimuli that afford only the relevant task. Human Brain Mapping, 33(5), 1137–1154. 10.1002/hbm.21277 21630376 PMC6870019

[bibr8-17470218241256361] Elchlepp H. Lavric A. Monsell S. (2015). A change of task prolongs early processes: Evidence from ERPs in lexical tasks. Journal of Experimental Psychology: General, 144(2), 299–325. 10.1037/a0038740 25844623

[bibr9-17470218241256361] Evans L. H. Herron J. E. Wilding E. L. (2015). Direct real-time neural evidence for task-set inertia. Psychological Science, 26(3), 284–290. 10.1177/0956797614561799 25626443 PMC4361352

[bibr10-17470218241256361] Faul F. Erdfelder E. Lang A. G. Buchner A. (2007). G*Power 3: A flexible statistical power analysis program for the social, behavioral, and biomedical sciences. Behavior Research Methods, 39, 175–191. 10.3758/BF03193146 17695343

[bibr11-17470218241256361] Forrest C. L. D. Monsell S. McLaren I. P. L. (2014). Is performance in task-cuing experiments mediated by task set selection or associative compound retrieval? Journal of Experimental Psychology: Learning, Memory, and Cognition, 40(4), 1002–1024. 10.1037/a0035981 24564543

[bibr12-17470218241256361] Geddert R. Egner T. (2022). No need to choose: Independent regulation of cognitive stability and flexibility challenges the stability-flexibility trade-off. Journal of Experimental Psychology: General, 151(12), 3009–3027. 10.1037/xge0001241 35617233 PMC9670017

[bibr13-17470218241256361] Goschke T. (2000). Intentional reconfiguration and involuntary persistence in task set switching. In Monsell S. Driver J. (Eds.), Control of cognitive processes: Attention and performance XVIII (pp. 331–355). The MIT Press.

[bibr14-17470218241256361] Goschke T. (2013). Volition in action: Intentions, control dilemmas, and the dynamic regulation of cognitive control. In Prinz W. Beisert M. Herwig A. (Eds.), Action science: Foundations of an emerging discipline (pp. 409–434). The MIT Press. 10.7551/mitpress/9780262018555.001.0001

[bibr15-17470218241256361] Green D. W. (1998). Mental control of the bilingual lexico-semantic system. Bilingualism: Language and Cognition, 1(2), 67–81. 10.1017/S1366728998000133

[bibr16-17470218241256361] Horoufchin H. Philipp A. M. Koch I. (2011). The dissipating task-repetition benefit in cued task switching: Task-set decay or temporal distinctiveness? Journal of Experimental Psychology: Human Perception and Performance, 37(2), 455–472. 10.1037/a0020557 20853997

[bibr17-17470218241256361] Hsieh S. Wu M. (2010). Age differences in switching the relevant stimulus dimensions in a speeded same-different judgement paradigm. Acta Psychologica, 135(2), 140–149. 10.1016/j.actpsy.2010.05.010 20598271

[bibr18-17470218241256361] Hsieh S. Wu M. (2011). Electrophysiological correlates of preparation and implementation for different types of task shifts. Brain Research, 1423, 41–52. 10.1016/j.brainres.2011.09.018 22000079

[bibr19-17470218241256361] Karayanidis F. Coltheart M. Michie P. T. Murphy K. (2003). Electrophysiological correlates of anticipatory and post-stimulus components of task switching. Psychophysiology, 40(3), 329–348. 10.1111/1469-8986.00037 12946108

[bibr20-17470218241256361] Kieffaber P. D. Hetrick W. P. (2005). Event-related potential correlates of task switching and switch costs. Psychophysiology, 42(1), 56–71. 10.1111/j.1469-8986.2005.00262.x 15720581

[bibr21-17470218241256361] Kieffaber P. D. Kruschke J. K. Cho R. Y. Walker P. M. Hetrick W. P. (2013). Dissociating stimulus-set and response-set in the context of task-set switching. Journal of Experimental Psychology: Human Perception and Performance, 39(3), 700–719. 10.1037/a0029545 22984990 PMC4427525

[bibr22-17470218241256361] Kiesel A. Steinhauser M. Wendt M. Falkenstein M. Jost K. Philipp A. M. Koch I. (2010). Control and interference in task switching—A Review. Psychological Bulletin, 136(5), 849–874. 10.1037/a0019842 20804238

[bibr23-17470218241256361] Kikumoto A. Hubbard J. Mayr U. (2016). Dynamics of Task-set Carry-over: Evidence from eye-movement analyses. Psychological Bulletin & Review, 23, 899–906. 10.3758/s13423-015-0944-y PMC4809786 26415999

[bibr24-17470218241256361] Koch I. (2001). Automatic and intentional activation of task sets. Journal of Experimental Psychology: Learning, Memory, and Cognition, 27, 1474–1486. 11713881 10.1037//0278-7393.27.6.1474

[bibr25-17470218241256361] Koch I. Kiesel A. (2022). Task switching: Cognitive control in sequential multitasking. In Kiesel A. Johannsen L. Koch I. Müller H. (Eds.), Handbook of human multitasking (pp. 85–143). Springer. 10.1007/978-3-031-04760-2_3

[bibr26-17470218241256361] Koch I. Lawo V. (2014). Exploring temporal dissipation of attention settings in auditory task switching. Attention, Perception, & Psychophysics, 76, 73–80. 10.3758/s13414-013-0571-5 24163154

[bibr27-17470218241256361] Koch I. Lawo V. Fels J. Vorländer M. (2011). Switching in the cocktail party: Exploring intentional control of auditory selective attention. Journal of Experimental Psychology: Human Perception and Performance, 37(4), 1140–1147. 10.1037/a0022189 21553997

[bibr28-17470218241256361] Koch I. Poljac E. Müller H. Kiesel A. (2018). Cognitive structure, flexibility, and plasticity in human multitasking—An integrative review of dual-task and task-switching research. Psychological Bulletin, 144(6), 557–583. 10.1037/BUL0000144 29517261

[bibr29-17470218241256361] Lavric A. Mizon G. A. Monsell S. (2008). Neurophysiological signature of effective anticipatory task-set control: A task-switching investigation. European Journal of Neuroscience, 28, 1016–1029. 10.1111/j.1460-9568.2008.06372.x 18717737

[bibr30-17470218241256361] Lee M. D. Wagenmakers E. (2013). Bayesian cognitive modeling: A practical course. Cambridge University Press. https://doi.org/10.1017/CBO9781139087759

[bibr31-17470218241256361] Liu C. Yeung N. (2020). Dissociating expectancy-based and experience-based control in task switching. Journal of Experimental Psychology: Human Perception and Performance, 46(2), 131–154. 10.1037/xhp0000704 31985251

[bibr32-17470218241256361] Loh K. Fels J. (2020). English speech material for a paradigm on intentional switching of auditory selective attention. Teaching and Research Area of Medical Acoustics, Institute of Technical Acoustics, RWTH Aachen University. 10.18154/RWTH-2020-08540

[bibr33-17470218241256361] Longman C. S. Elchlepp H. Monsell S. Lavric A. (2021). Serial or parallel proactive control of components of task-set? A task-switching investigation with concurrent EEG and eye-tracking. Neuropsychologia, 160, Article 107984. 10.1016/j.neuropsychologia.2021.107984 34339718

[bibr34-17470218241256361] Longman C. S. Lavric A. Monsell S. (2016). The coupling between spatial attention and other components of task-set: A task-switching investigation. Quarterly Journal of Experimental Psychology, 69, 2248–2275. 10.1080/17470218.2015.1115112 27033987

[bibr35-17470218241256361] Longman C. S. Lavric A. Monsell S. (2017). Self-paced preparation for a task switch eliminates attentional inertia but not the performance switch cost. Journal of Experimental Psychology: Learning, Memory, and Cognition, 43, 862–873. 10.1037/xlm0000347 27936844

[bibr36-17470218241256361] Longman C. S. Lavric A. Munteanu C. Monsell S. (2014). Attentional inertia and delayed orienting of spatial attention in task-switching. Journal of Experimental Psychology: Human Perception and Performance, 40, 1580–1602. 10.1037/a0036552 24842065

[bibr37-17470218241256361] Mayr U. Kuhns D. Rieter M. (2013). Eye movements reveal dynamics of task control. Journal of Experimental Psychology: General, 142(2), 489–509. 10.1037/a0029353 22844987

[bibr38-17470218241256361] Meiran N. (1996). Reconfiguration of processing mode prior to task performance. Journal of Experimental Psychology: Learning, Memory, and Cognition, 22, 1423–1442. 10.1037/0278-7393.22.6.1423

[bibr39-17470218241256361] Meiran N. (2000). Modeling cognitive control in task-switching. Psychological Research, 63, 234–249. 10.1007/s004269900004 11004878

[bibr40-17470218241256361] Meiran N. Chorev Z. Sapir A. (2000). Component processes in task switching. Cognitive Psychology, 41(3), 211–253. 10.1006/cogp.2000.0736 11032657

[bibr41-17470218241256361] Meiran N. Kessler Y. Adi-Japha E. (2008). Control by action representation and input selection (CARIS): a theoretical framework for task switching. Psychological Research, 72, 473–500. 10.1007/s00426-008-0136-8 18350316

[bibr42-17470218241256361] Meiran N. Marciano H. (2002). Limitations in advance task preparation: Switching the relevant stimulus dimension in speeded same-different comparisons. Memory & Cognition, 30(4), 540–550. 10.3758/BF03194955 12184555

[bibr43-17470218241256361] Milne A. E. Bianco R. Poole K. C. Zhao S. Oxenham A. J. Billig A. J. Chait M. (2021). An online headphone screening test based on dichotic pitch. Behavior Research Methods, 53, 1551–1562. 10.3758/s13428-020-01514-0 33300103 PMC7725427

[bibr44-17470218241256361] Monsell S. (2003). Task switching. Trends in Cognitive Sciences, 7, 134–140. 10.1016/S1364-6613(03)00028-7 12639695

[bibr45-17470218241256361] Monsell S. (2015). Task-set control and task switching. In Fawcett J. Risko E. F. Kingstone A. (Eds.), The handbook of attention (pp. 139–172). The MIT Press. 10.1093/med:psych/9780198528883.003.0002

[bibr46-17470218241256361] Monsell S. (2017). Task set regulation. In Egner T. (Ed.), The Wiley handbook of cognitive control (pp. 29–49). John Wiley & Sons. 10.1002/9781118920497.ch2

[bibr47-17470218241256361] Monsell S. Lavric A. Strivens A. Paul E. (2019). Can we prepare to attend to one of two simultaneous voices? Journal of Experimental Psychology: Human Perception and Performance, 45(7), 966–982. 10.1037/xhp0000650 31021156

[bibr48-17470218241256361] Monsell S. Mizon G. A. (2006). Can the task-cuing paradigm measure an endogenous task-set reconfiguration process? Journal of Experimental Psychology: Human Perception and Performance, 32(3), 493–516. 10.1037/0096-1523.32.3.493 16822121

[bibr49-17470218241256361] Monsell S. Sumner P. Waters H. (2003). Task-set reconfiguration with predictable and unpredictable task switches. Memory & Cognition, 31, 327–342. 10.3758/BF03194391 12795475

[bibr50-17470218241256361] Musslick S. Cohen J. D. (2021). Rationalizing constraints on the capacity for cognitive control. Trends in Cognitive Sciences, 25(9), 757–775. 10.1016/j.tics.2021.06.001 34332856

[bibr51-17470218241256361] Musslick S. Jang S. J. Shvartsman M. Shenhav A. Cohen J. D. (2018). Constraints associated with cognitive control and the stability-flexibility dilemma. In Rogers T. T. Rau M. Zhu X. Kalish C. W. (Eds.), Proceedings of the 40th annual meeting of the Cognitive Science Society (pp. 804–809). Cognitive Science Society.

[bibr52-17470218241256361] Nolden S. Koch I. (2017). Intentional switching of auditory attention between long and short sequential tone patterns. Attention, Perception, & Psychophysics, 79, 1132–1146. 10.3758/s13414-017-1298-5 28205053

[bibr53-17470218241256361] Nolden S. Koch I. (2023). Preparing auditory task switching in a task with overlapping and non-overlapping response sets. Psychological Research, 87, 2228–2237. 10.1007/s00426-023-01796-x PMC10457221 36790482

[bibr54-17470218241256361] Rogers R. D. Monsell S. (1995). Costs of a predictable switch between simple cognitive tasks. Journal of Cognitive Psychology: General, 124, 207–231. 10.1037/0096-3445.124.2.207

[bibr55-17470218241256361] Rushworth M. F. S. Passingham R. E. Nobre A. C. (2002). Components of switching intentional set. Journal of Cognitive Neuroscience, 14(8), 1139–1150. 10.1162/089892902760807159 12495521

[bibr56-17470218241256361] Rushworth M. F. S. Passingham R. E. Nobre A. C. (2005). Components of attentional set-switching. Experimental Psychology, 52(2), 83–98. 10.1027/1618-3169.52.2.83 15850156

[bibr57-17470218241256361] Siqi-Liu A. Egner T. (2020). Contextual adaptation of cognitive flexibility is driven by task- and item-level learning. Cognitive, Affective, & Behavioral Neuroscience, 20, 757–782. 10.3758/s13415-020-00801-9 PMC7396276 32495271

[bibr58-17470218241256361] Siqi-Liu A. Egner T. Woldorff M. G. (2022). Neural dynamics of context-sensitive adjustments in cognitive flexibility. Journal of Cognitive Neuroscience, 34, 480–494. 10.1162/jocn_a_01813 35015871 PMC9403815

[bibr59-17470218241256361] Steinhauser M. Hübner R. (2009). Distinguishing response conflict and task conflict in the Stroop task: Evidence from ex-Gaussian distribution analysis. Journal of Experimental Psychology: Human Perception and Performance, 35(5), 1398–1412. 10.1037/a0016467 19803645

[bibr60-17470218241256361] Strivens A. Koch I. Lavric A. (2024). Does preparation help to switch auditory attention between simultaneous voices: Effects of switch probability and prevalence of conflict. Attention, Perception, & Psychophysics. Advance online publication. 10.3758/s13414-023-02841-y PMC11062987 38212478

[bibr61-17470218241256361] Van’t Wout F. Lavric A. Monsell S . (2015). Is it harder to switch among a larger set of tasks? Journal of Experimental Psychology: Learning, Memory, and Cognition, 41(2), 363–376. 10.1037/a0038268 25528089

[bibr62-17470218241256361] Yeung N. Nystrom L. E. Aronson J. A. Cohen J. D. (2006). Between-task competition and cognitive control in task switching. Journal of Neuroscience, 26(5), 1429–1438. 10.1523/JNEUROSCI.3109-05.2006 16452666 PMC6675498

